# Dynamic Nuclear Polarization NMR as a new tool to investigate the nature of organic compounds occluded in plant silica particles

**DOI:** 10.1038/s41598-017-03659-z

**Published:** 2017-06-13

**Authors:** Armand Masion, Anne Alexandre, Fabio Ziarelli, Stéphane Viel, Guaciara M. Santos

**Affiliations:** 1 CNRS, Aix-Marseille Université, CEREGE, IRD, 13545 Aix en Provence, France; 2 Labex Serenade, 13545 Aix en Provence, France; 3 Aix-Marseille Université, CNRS, Centrale Marseille, Fédération Sciences Chimiques Marseille, 13397 Marseille, France; 4 Aix-Marseille Université, CNRS, ICR, 13397 Marseille, France; 5 Institut Universitaire de France, 75005 Paris, France; 6 Earth System Science, University of California, Irvine, USA

## Abstract

The determination of the chemical nature of the organic matter associated with phytoliths remains a challenge. This difficulty mainly stems from amounts of organic carbon (C) that are often well below the detection limit of traditional spectroscopic tools. Conventional solid-state ^13^C Nuclear Magnetic Resonance (NMR) is widely used to examine the nature and structure of organic molecules, but its inherent low sensitivity prohibits the observation of diluted samples. The recent advent of commercial microwave source in the terahertz range triggered a renewed interest in the Dynamic Nuclear Polarization (DNP) technique to improve the signal to noise ratio of solid-state NMR experiments. With this technique, the ^13^C spectrum of a phytolith sample containing 0.1% w/w C was obtained overnight with sufficient quality to permit a semi-quantitative analysis of the organic matter, showing the presence of peptides and carbohydrates as predominant compounds. Considering the natural abundance of the ^13^C isotope, this experiment demonstrates that DNP NMR is sufficiently sensitive to observe spin systems present in amounts as low as a few tens of ppm.

## Introduction

The molecular analysis of natural organic matter in organisms, soils or sediments is a challenging field for our understanding of the processes involved in the carbon cycle. Among the many challenges to be met is the difficulty in obtaining a quantitative or semi-quantitative speciation of the organic carbon, with the least possible preparation steps. Indeed, several chemical steps are often required to purify the molecular compounds to be analyzed, from the minerals they are associated with^1–3^. Unfortunately these steps can modify the molecular nature of the compounds to be analyzed, especially when HF is involved^4^. In this context, ^13^C Nuclear Magnetic Resonance (NMR), is an attractive method since it is element specific and therefore does not necessarily require prior extraction of the organic C, as long as the mineral matrix is free from paramagnetic elements (e.g. Fe(II), Fe(III), Cu(II), etc.) which cause substantial signal loss by broadening the lines beyond detection^5–7^. Furthermore, ^13^C NMR provides a detailed analysis of the nature of the organic C from its surrounding matrix, and the quantitative exploitation can be easily performed using an internal or external reference, or without calibration when only relative proportions are needed. This technique has been successfully used to investigate plant tissuese.g^8^. and transformation of organic matter in soils e.g. refs 9–11. In this context, ^13^C NMR analysis of organics associated with biogenic silica is a favorable case. In higher plants, silicon is acquired by roots from soils and precipitated in or between the cells as micrometric hydrous amorphous biosilica particles called phytoliths. Phytolith abundances range from <1% of dry weight (dwt) in many plants to several % dwt in grasses that are Si-accumulators^12, 13^. Phytoliths contain small amounts (<0.5% of dry weight) of carbon (C) occluded during silica precipitation^14–17^, commonly termed as phytC. Recently, the phytC content, nature, origin and impact in the global C cycle have become the subject of increasing debate^14, 15, 18–23^. Based on the assumptions that phytC is of photosynthetic origin and is preserved from mineralization in soils, claims were recently made that phytoliths from several agriculturally important monocotyledonous species play a significant role in atmospheric CO_2_ sequestration^14, 15, 18–23^. However, comparative isotopic measurements (^14^C and δ^13^C) of phytC, plant tissues, atmospheric CO_2_, and soil organic matter recently showed that phytC is partially derived from soil carbon, raising the question of the relevance of phytC as a significant sink of atmospheric CO_2_^14, 15^. Additionally, three-dimensional X-ray microscopy and nanoscale secondary ion mass spectrometry (NanoSIMS) analyses suggested that phytC consists of two pools of C, protected differently from mineralization by the silica structure, questioning the hypothesis of long persistence of phytC in soils^18^. This set of studies additionally called for molecular characterization of phytC to better understand its origin, occlusion and resistance to weathering, and properly quantify all fluxes involved in the phytolith carbon cycle^14, 15^.

Several techniques have been used to characterize phytC (e.g. high-performance liquid chromatography, gas chromatography mass spectrometry, protein staining, micro-Raman analysis or X-ray photoelectron spectroscopy) and led to contradictory results, especially regarding the presence or not of amino acids^14, 15^. However, the validity of those analyses cannot be granted, as evaluations used phytolith concentrates that were not necessarily proven to be completely devoid of extraneous organic remains.

In this context, a non-destructive analytical technique such as NMR constitutes a real asset because it can be used to determine the C speciation without any preliminary dissolution of silica. However, even in favorable cases, the inherent lack of sensitivity of NMR remains a major limitation. This is especially true for carbon whose NMR-active isotope is only approx. 1.1% abundant, which gives overall a ^13^C NMR receptivity that is about 5000 times lower than for ^1^H. In addition, recycle delays between each acquisition (or scan) are typically (much) longer for ^13^C than for ^1^H, which further increases the data collection time to get a spectrum with a sufficient signal-to-noise ratio (S/N).

Hardware improvements contribute to increase the S/N. For example, the use of cryoprobes in liquid-state NMR can lead to up to 16 times shorter data acquisition times. For solid-state NMR experiments, the improvement of S/N is often achieved by cross polarization (CP), i.e. the magnetization transfer from an abundant spin (e.g. ^1^H) to a dilute spin (e.g. ^13^C or ^29^Si). In the specific case of natural organics, the theoretical gain of a ^1^H-^13^C cross-polarization magic angle spinning (CPMAS) experiment is approx. 4 (related to γ_H-1_/γ_C-13_, where γ is the gyromagnetic ratio of the nucleus). In practice, the gain in S/N is usually better since faster ^1^H recycle delays allow for more scans to be collected per unit time. However, when the weight percentage of carbon within the sample drops into the single digit range and/or the total sample amount is limited, even the accumulation of several thousands of scans may not produce a sufficient S/N.

Polarization cannot only be transferred from one nuclear spin system to another, but also from an electron to a nuclear spin system, also known as Dynamic Nuclear Polarization (DNP). The unpaired electron can be internal to the sample, or, provided by doping with a paramagnetic substance (typically a dinitroxide)^24^. In this case, the polarization transfer is achieved by high-power microwave irradiation of the sample at or near the electron paramagnetic resonance transition, usually at cryogenic temperatures (around 100 K) in order to increase the electron and nuclear relaxation times. The benefit is the large theoretical gain based on the gyromagnetic ratios, viz. approx. 660 for ^1^H and 2600 for ^13^C. The feasibility of this method was demonstrated in the 1950s at field strength below 1 T^25, 26^. But during the following decades, the improvement of the NMR S/N was primarily achieved by the development of affordable high-field magnets. Erroneous predictions on the high-field utility of DNP, which were later disproven, led to a latent period in its development. In the 1990s, the development of suitable microwave sources in the near THz range made possible the use of DNP at high magnetic fields (>5 T)^27–29^. This triggered a renewed interest in the DNP technique and made it available to a larger audience, and especially the biology- and material science communities^30–32^. However, the literature reveals that its application to environmental samples remains very limited.

The potential of DNP NMR to observe organic ligands associated with silicate particles has been demonstrated recently. The weight percentage of carbon present within the sample varied from ca. 15% for protein analysis on diatoms down to 0.1% to examine the binding of organic hydration inhibitors to cementitious tricalcium silicate phases^33, 34^. In these studies, the detection of carbon was facilitated by substantial, i.e. up to 99%, ^13^C enrichment of the organic ligand. Of course, no such isotopic enrichment can be expected in natural samples. In the present study, we demonstrate that DNP NMR can produce insightful spectra for low carbon concentration of phytolith extracts at natural ^13^C abundance.

## Phytolith extraction

**Figure 1:**
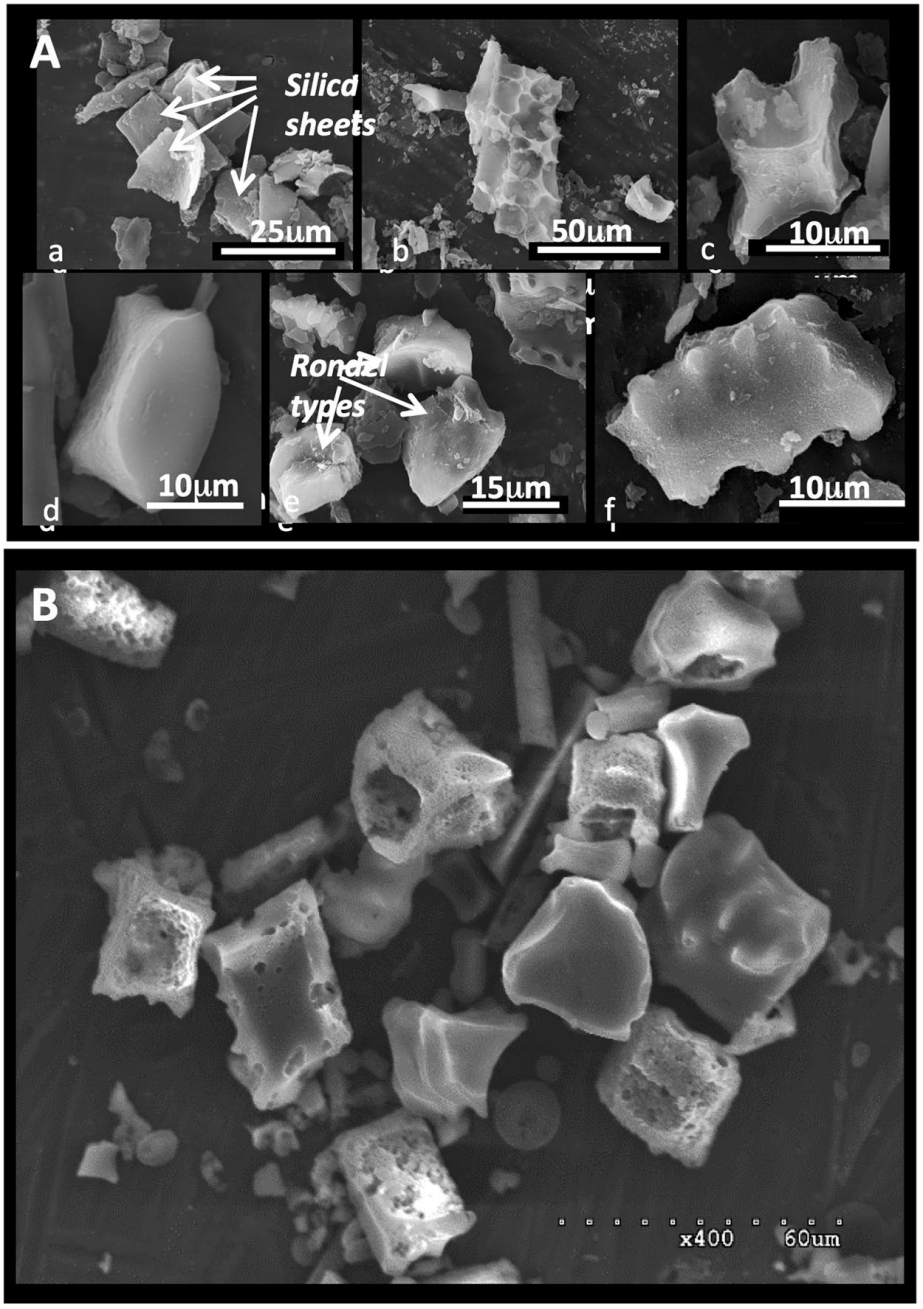
From Alexandre *et al*. Biogeosciences (2015) (ref. 18.) SEM images of (**A**) TD-F-L wheat phytolith assemblage. Three categories are illustrated: (1) silica sheets (a,b), (2) stellate type from intercellular space (c) and (3) GSC phytoliths including rondel (d,e) and polylobate types (f); and (**B**) MSG70 weathered soil phytoliths. The particles show enlarged internal cavities typical of weathered phytoliths.

Two grass phytolith samples were analyzed. The first sample (TD-F-L) was extracted from the leaves of Triticum durum wheat harvested in 2012 at the Genomics Research Centre in Fiorenzuola d’Arda (Italy), using a wet chemical protocol described previously, specifically designed for reliable isotopic analyses of phytC^35^. The plant organic matter was oxidized at 80 °C with H_2_SO_4_, H_2_O_2_, HNO_3_ and KClO_3_, and alkaline-soluble forms of the organic matter on the phytolith surface were dissolved using KOH (pH 11). The short duration of this treatment (10 min) warrants a negligible dissolution of the silica (ca. 10^−6^ times the initial SiO_2_ mass)^36^. The absence of residual extraneous organic particles was checked using SEM-EDX^15^. The phytolith morphological assemblage was observed in natural light microscopy (Fig. 1A)^18^. Grass short cell (GSC) particles from the sample were also analyzed using 3-D X-ray microscopy, which displayed the presence of internal cavities closed or open to the surface by micrometric connections^18^. Additionally, nanoSIMS analyses of the GSC particles displayed a pool of phytC homogeneously distributed in the silica structure. TD-F-L phytC and nitrogen (phytN) contents, measured by chemiluminescence after combustions, are respectively 0.4 and 0.1% dry weight^18^. When measured by Isotope-ratio mass spectrometry (IRMS) and after sealed-tube combustion at 900 °C prior to ^14^C analysis the phytC content is 0.09% by dry weight in average^15^.

The second sample (MSG70) was extracted from a Mascareignite soil (La Réunion island, France) as described previously^37^, using HCl, C_6_H_5_Na_3_O_7_, Na_2_O_4_S_2_, H_2_O, H_2_O_2_, Na(PO_3_)_6_ and ZnBr_2_ at a temperature of 70 °C. The phytolith extract contain highly weathered fossil grass phytoliths of approximately 2.5ka yrs BP (uncalibrated ^14^C age) in average^16^. Most of this phytolith extract is constituted of hollow particles (Fig. 1B). MSG70 phytC analyzed after sealed-tube combustion at 900 °C prior to ^14^C measurement also accounts for 0.09% by dry weight on average^16^, similarly to TD-F-L. However, thermograms obtained for both MSG70 and TD-F-L using oxidation reactivity on a modified Thermal-Optical Carbon Aerosol Analyzer (RT 3080, Sunset Laboratory Inc.) showed an overall production of CO_2_ much lower for MSG70 than for TD-F-L^16^, suggesting a significant difference in phytC concentration. Although our % quantification of phytC from phytolith extracts has been highly reproducible when using the inner calibrated-volume of the vacuum line after phytolith sealed-tube combustion at 900 °C and/or IRMS measurements^15, 16^, as a side note the modified Thermal-Optical Carbon Aerosol Analyzer is by far more sensitive to detect lower carbon percentage levels^38^.

## DNP NMR analysis of the phytoliths

The solid-state DNP CPMAS experiments described in this work were recorded on a Bruker NMR spectrometer operating at 9.4 T (400 MHz for the ^1^H Larmor frequency) controlled by an AVANCE-III console and equipped with a 3.2 mm low-temperature DNP ^1^H/X/Y magic-angle spinning (MAS) probe manufactured by Bruker. This spectrometer was equipped with a gyrotron that provided microwave (*μ*w) irradiation of the sample. Specifically, the field sweep coil of the NMR magnet was set so that *μ*w irradiation occurred at the maximum DNP enhancement of TOTAPOL (263.334 GHz)^39^. The estimated power of the *μ*w beam at the output of the probe waveguide was ~5 W. The sample temperature (with *μ*w switched on) was ~105 K with a MAS speed of 10 kHz. During the CPMAS experiments, the *μ*w irradiation field was turned on. Moreover, the amplitude of the ^1^H contact pulse (2.5 ms duration) in CPMAS experiments was linearly ramped in order to improve CP efficiency^40^. The DNP polarizing radical used in this study was a dinitroxide known as AMUPol^41^. This polarizing agent exhibits good water solubility. The TD-F-L and MSG70 samples for DNP were prepared according to the so-called incipient wetness impregnation method^42^ by wetting 20 mg of sample in a watch glass with 20 *μ*L of a 10 mM aqueous solution of AMUPol. The samples were then stirred with a glass rod to homogeneously wet the solid. This step worked satisfactorily with the TD-F-L phytoliths, but resulted in a somewhat heterogeneous mixture with MSG70. To improve the wetting of the solid, the AMUPol biradical was also added in DMSO-*d*_6_/H_2_O (60/40, v/v) and in the so-called ‘DNP juice’, which consists of glycerol/D_2_O/H_2_O (60/30/10, v/v/v) using ^13^C-depleted solvents. The resulting partially wet solids (approx. 30 mg) were eventually transferred into a 3.2 mm (o. d.) MAS sapphire rotor containing a Teflon insert and capped with a zirconium drive cap. ^13^C chemical shifts were externally referenced with respect to tetramethylsilane. Free Induction Decays were processed using the MestReNova software (processing included 50 Hz line broadening, Fourier transform, phase and baseline corrections). Spectra were line fitted using the Igor Pro software package.

## Speciation of the carbon occluded within the phytoliths

About 20 mg of each phytolith material were analyzed using solid-state DNP NMR. Given the phytC contents of 0.09–0.1% dry weight and a natural abundance of 1.07% for the ^13^C isotope, this corresponds to a weight concentration of the ^13^C spin system in the sample of only 10 ppm.

**Figure 2:**
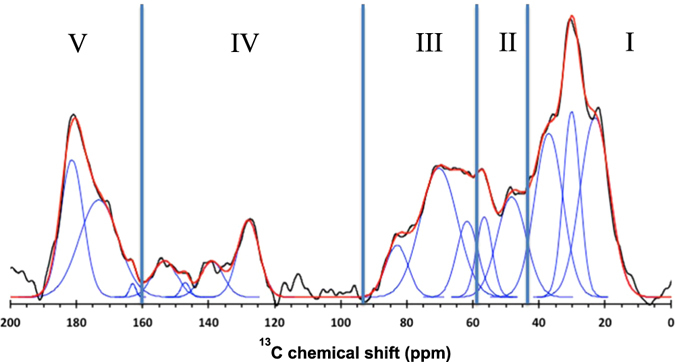
DNP ^1^H-^13^C CP-MAS spectrum of the TD-F-L phytolith sample (black) and its line fitting (red) with Gaussian peaks (blue), Roman numerals refer to the main chemical categories of Table 1.

The CPMAS spectrum of the TD-F-L phytolith sample is displayed in Fig. 2. It is the result of the acquisition of 35664 scans with microwaves on, obtained in less than 15 hours. We estimate that the spectrum of equivalent quality without microwaves and at room temperature would have required almost a year. Although the S/N is rather limited, the so-obtained data is still of sufficient quality for a semi-quantitative analysis of the organic matter occluded within the phytoliths. The detected signal covers the entire chemical shift range of ^13^C, i.e. from 0 to 200 ppm. The overall shape of the spectrum is typical of natural organics with contribution from all major functional group categories, viz. alkyl (0–30 ppm), alkyl-N (40–60 ppm), carbohydrates (60–100 ppm), aryl (110–150 ppm), and carbonyl (160–190 ppm). These categories correspond respectively to the spectral regions I through V in Table 1 and Fig. 2. The relative proportion of each of these categories was determined by line fitting the spectrum with Gaussian peaks. No constraints were applied to the peaks parameters, and in particular peak width, to allow for minimization of the fit residue. Consequently, the carbon speciation determined on a peak-by-peak analysis would bear only limited relevance. However, although the resolution of the present data precludes the identification of specific compounds, the speciation by grouping contributions into generic chemical categories remains a major step forward for the analysis of natural organics given the low concentration of C within the pure phytolith extract.

**Table 1:** Proportions of the main chemical categories of phytC occluded within the TD-F-L phytolith.

	ppm	%
Zone I alkyl	23.0	37
	30.1	
	37.0	
Zone II alkyl-N	48.3	12
	56.6	
Zone III alkyl-O	61.7	21
	70.3	
	83.0	
Zone IV aryl	127.9	10
	139.0	
	147.1	
	153.3	
Zone V carbonyl	163.0	20
	173.3	
	181.4	

Within the phytolith sample, over 1/3 of the signal corresponds to alkyl carbon (Table 1). The contribution between 40 and 60 ppm, assigned to nitrogenated carbon (amines, amides) represents ca. 10%, strongly suggesting the presence of amino-acids and proteins. This region overlaps with the carbohydrate contribution which accounts for approx. 20% of the carbon (Table 1). With a 1:4:1 surface ratio for the contributions at ca. 60, 70 and 80 ppm, the general aspect of the spectrum in this range is consistent with saccharidic compounds, without, however, an unequivocal detection of anomeric carbon due to the insufficient S/N. With a proportion of only 10% (Table 1), aromatic compounds are minor C species. These contributions probably correspond to amino-acid side chains (histidine, phenylalanine, triptophan, tyrosine) and lignins. Finally, the carbonyls account for ca. 20% of the carbon within the sample. In a first approximation, this contribution corresponds to peptide bonds in proteins and carboxyls from uronate moieties in carbohydrate fraction of the organic carbon.

In contrast with TD-F-L, no usable ^13^C NMR signal could be obtained for the fossil MSG70 phytolith sample. Modifying the composition of the wetting solution and increasing the number of scans beyond 70000 did not result in any improvement of the signal.

## Discussion

The absence of ^13^C NMR signal detection for the MSG70 phytoliths is most likely a combination of several factors. The first parameter is its lower carbon concentration evidenced by the thermograms obtained by oxidation reactivity in the modified Thermal-Optical Carbon Aerosol Analyzer^15^. Although both phytolith extracts produced CO_2_ following a similar temperature ramp profile, the overall production of phytC CO_2_ for MSG70 was lower by approx. 43% when compared to the amount obtained by the fresh leaf phytolith extract. This may correspond to phytC a concentration below the detection limit of DNP NMR.

However the predominant cause for the absence of signal is probably the low specific surface area (SSA) of MSG70 phytolith extract. Indeed, we performed standard N_2_ BET analyses on both phytoliths (using a Micromeritics ASAP2020 instrument). The TD-F-L sample had a SSA of 124.9 m^2^/g which is of the order of previous findings with the same type of materials^43^, whereas the measured SSA for the MSG70 was only 2.2 m^2^/g. Although counterintuitive, this drastic reduction of the SSA of aged/weathered silica is well documented^44–48^, including for phytolith material^49^.

In terms of DNP-NMR spectroscopy, the polarization transfer is of course strongly limited by the reduction of the SSA by two orders of magnitude, especially since, in the case of silica, this is often accompanied by dehydroxylation^46, 48^, which could further reduce the polarization transfer efficiency. This may explain why trying various radical-containing impregnating solutions remained unsuccessful. These drastic detrimental effects associated with a lower C content in the fossil phytoliths puts the carbon speciation in this material out of the reach of the capabilities of current DNP-NMR spectroscopy.

The composition of the organics in the TD-F-L phytolith sample clearly demonstrates the variety of carbon compounds, suggesting the possibility of different origins. The presence of alkyl, alkyl-N and carbonyls are consistent with a significant proportion of proteins. Given a proportion of about 10% for the C-N compounds (Table 1) and an average of 5–6 C atoms per amino acid, half of the detected NMR signal would correspond to peptides, i.e. well beyond typical protein proportions for wheat leaf tissue (or straw) e.g. refs 50 and 51. This shows that the PhytC speciation is not simply inherited from the composition of the host leaf. It has been demonstrated from ^14^C and ^13^C labeling that some of the carbon occluded in phytoliths can derived from soil solution and probably from proteins and/or amino acids^52–55^ commonly absorbed by plant roots^52–55^. However it was not possible to identify the form in which this soil C is ultimately fixed in phytoliths. The high proportion in amino compounds evidenced in the present study supports that the (poly)peptides associated with the silica are partly imported from compartments outside the silicified plant cells.

With regard to the carbohydrate signal, it is reasonable to assign it to photosynthetic carbon produced by the host leaf, i.e. saccharides and their degradation products such as pyruvate and ATP, rather than to mucilage-type glycoproteins.

The carbon speciation described in the present study from DNP NMR for the TD-F-L phytolith is very different from the phytC composition of Triodia obtained from traditional CPMAS NMR^56^. Indeed the Trodia phytC was described as consisting predominantly of saccharides and about 1/3 of alkyl C not assigned to any particular category of compounds^56^, whereas the TD-F-L phytC analyzed in the present study has a more varied and complex composition with marked contributions of peptides and carbonyls in addition to the alkyl-C and carbohydrate signals (Table 1, Fig. 2). This difference is most likely due to the NMR sample preparation: the TD-F-L phytolith material was analyzed as is, whereas for Trodia the phytC was extracted using HF. This treatment is known to cause significant loss of organic C and is prone to artifacts (modification of the chemical nature)^57–61^.

With the current data, it is not possible to discuss the carbon speciation in any more detail without becoming speculative. At comparable concentrations, more detailed information regarding the nature of organic carbon involves pyrolysis and chromatographic tools, i.e. destructive methods with possible matrix interaction- and sample preparation biases. However, although this level of detail is rather coarse for NMR standards, the considerable gain in sensitivity associated with the use of DNP now makes NMR a relevant technique for the analysis of environmental samples since spin systems as dilute as a few tens of weight ppm can be examined under reasonable conditions.
